# Medical care services provision and stress experience in urologists during all waves of the COVID-19 pandemic in Germany

**DOI:** 10.3389/fmed.2024.1320489

**Published:** 2024-02-09

**Authors:** Pia Paffenholz, Moritz Platen, Karel Kostev, Sven H. Loosen, Jens Bohlken, Bernhard Michalowsky

**Affiliations:** ^1^Department of Urology, Uro-Oncology, Robot Assisted and Reconstructive Urologic Surgery, University of Cologne Faculty of Medicine and University Hospital Cologne, Cologne, Germany; ^2^German Center for Neurodegenerative Diseases (DZNE) Site Rostock/Greifswald, Ellernholzstrasse 1-2, Greifswald, Germany; ^3^Epidemiology, IQVIA, 60549, Frankfurt, Germany; ^4^Clinic for Gastroenterology, Hepatology and Infectious Diseases, University Hospital Düsseldorf, Medical Faculty of Heinrich Heine University Düsseldorf, Düsseldorf, Germany; ^5^Occupational Medicine, and Public Health (ISAP) of the Medical Faculty at the University of LeipzigInstitute for Social Medicine, Leipzig, Germany

**Keywords:** ambulatory health care system, cancer, COVID-19 pandemic, mental health, SARS-CoV2, tele medicine

## Abstract

**Purpose:**

Urologists’ practices reported decreasing medical care provision and increasing stress experience in the first wave of the COVID-19 pandemic. However, long-term effects of the pandemic are unknown.

**Methods:**

Medical record data of n = 127 urologists were used to assess changes in healthcare provision, comparing the pandemic with the pre-pandemic period. An online survey among n = 101 urologists was conducted to assess the physicians’ perceptions of the identified healthcare provision and organizational changes and experiences of anxiety, stress, and support needs during the pandemic waves. Urologists consultations, specialists’ referrals, hospital admissions, documented cancer diagnoses, urologists’ perceptions of causes for these changes and experienced stress, anxiety and support needs. Results were demonstrated using descriptive statistics.

**Results:**

Over the first two years of the pandemic, there was a slight decline in consultations (−0,94%), but more intensive reduction in hospital admissions (−13,6%) and identified cancer diagnoses (−6,2%). Although patients’ behavior was seen as the main reason for the changes, 71 and 61% of consultations of high-risk patients or urgent surgeries were canceled. Telemedical approaches were implemented by 58% of urologists, and 88% stated that the reduced cancer detection rate would negatively affect patients’ outcomes. Urologists reported higher anxiety, stress, and need for support during all waves of the pandemic than other disciplines, especially females.

**Conclusion:**

The pandemic tremendously affects urologists’ health care provision and stress experience, possibly causing long-term consequences for patients and physicians.

## 1 Introduction

As the COVID-19 pandemic led to a global crisis, contact restrictions were imposed, and medical measures were reduced to the minimum to contain the virus’ rapid dissemination (1, 2). These restrictions did not only concern hospital staff, increasing intensive care capacities and postponing elective surgical procedures, but the outpatient sector was also intensively affected (3–5).

A cross-sectional study based on nearly four million consultations evaluated the number of healthcare services during the first pandemic wave, demonstrating a 65% decline in face-to-face consultations (6). The healthcare services provided in the ambulatory sector also significantly decreased during the first wave, especially when the protective measures were imposed in March 2020, affecting mainly elective consultations (7).

At the beginning of the pandemic, urologists from the outpatient sector were not adequately prepared to deal with the new situation in their practices, causing considerable concern and fear among physicians (8–10). During the first COVID-19 wave, 24% of all urologists felt high, and 48% felt a moderate threat level (8). A German observational study revealed that hospital admissions, recognized incident diseases, and consultations significantly decreased throughout the pandemic in primary care (11). However, it is unknown whether and, if so, to what extent the reduced provision of urologist services was compensated at the end of the pandemic.

The negative impact of the pandemic on the mental health of healthcare professionals strongly influences work-life (12). A prior survey- and interview-based study revealed significant physical and psychological burden associated with the pandemic, with a high prevalence of burnout (57.7%) among physicians (13). Especially studies from Asia demonstrated high stress, depression, and anxiety symptoms with severe degrees in 2.2 to 14.5% at the pandemic’s beginning (14). However, data are lacking regarding the stress experienced by urologists during the pandemic.

This analysis’s objectives were to evaluate the impact of the COVID-19 pandemic on the daily work routine and medical service provision of urologists and their perceived stress during the first four pandemic waves in Germany.

## 2 Materials and methods

### 2.1 Study design

This study comprised a secondary data analysis based on medical record data from the Disease Analyzer database (IQVIA) to examine changes in healthcare service provision and a survey to assess perceived reasons for these changes. The secondary data captured consultations, drug prescriptions, specialist referrals, diagnoses made, and basic medical and demographic data directly and anonymously from the practices via an interface to their respective practice management software between September 2019 and February 2020 (pre-pandemic period) as well as March 2020 and September 2021 (pandemic period). The survey questionnaire was distributed using the cloud-based open-source tool LimeSurvey. The Professional Association of German Urologists e.V shared the survey link between 04 December 2021 and 28 February 2022 with their members via different communication channels. All participants agreed on the conditions of the survey before taking part. The survey was approved by the Ethical Committee of the Chamber of Physicians of Mecklenburg-Western Pomerania registry number (BB 127/21). Detailed information is given in the supplementary material.

### 2.2 Statistical analyses

Descriptive statistics were used to demonstrate changes in healthcare provision, recognition of cancer cases and perception and views on causes of these changes. Fisher’s exact Tests were used to check significance of these differences. Multivariate regression models were used to assess associated factors of stress, anxiety and support needed of urologists. Analyses were performed using SAS version 9.4 (Cary, NC: SAS Institute Inc) and STATA/IC 16.

## 3 Results

### 3.1 The course of consultations, drug prescriptions, hospital admissions, and incident cancer diagnoses

**Figure 1:**
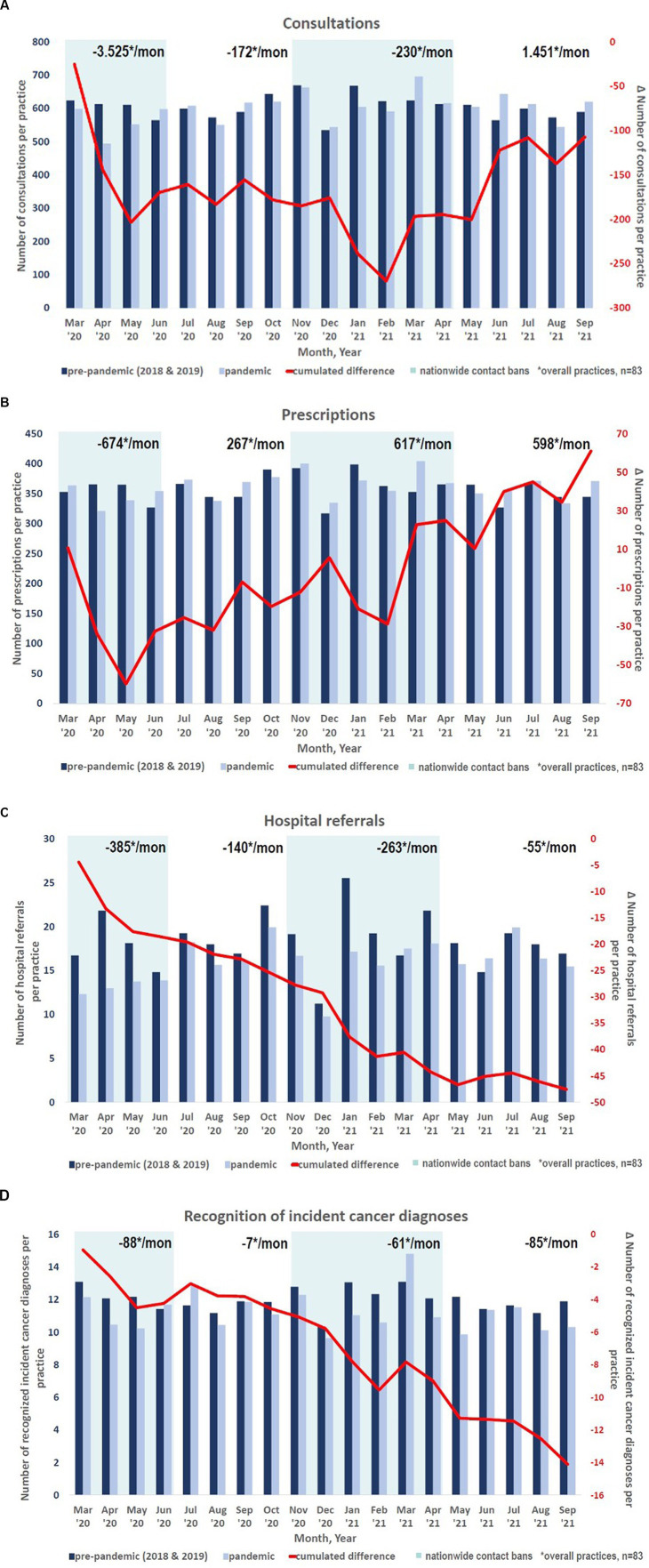
**(A,B)** Trends in consultation frequency, drug prescriptions, hospital admissions and cancer diagnosis over the pandemic periods. Number of the examined parameter per practice is displayed on the y-axis on the left. The dark blue column indicates the reference value (average of the respective month of the years 2018/19), the light blue column shows the value in the respective month of the pandemic (2020/21). The phases of the federal contact restriction measures are indicated by two large turquoise rectangles, an average value was calculated for all phases. The red line and the y-axis on the right display the cumulative difference (%) of the target value during the pandemic compared to the pre-pandemic month. **(A)** Trends in consultation frequency over the pandemic periods. **(B)** Trends in drug prescriptions over the pandemic periods. **(C)** Trends in hospital admissions over the pandemic periods. **(D)** Trends in incident cancer diagnoses over the pandemic periods.

During the 1st COVID-19 wave (March to June 2020), 11% fewer consultations per month were seen compared to the same period in 2018 and 2019. During the subsequent waves, consultations did not reach the frequency of the corresponding period the year before the pandemic (−1%) and decreased even to a greater extent with the implementation of contact restrictions in February 2021 (−3,4%). From May to September 2021 (3rd wave and summer plateau), the consultation frequency increased again but did not reach the values of the pre-pandemic years 2018 and 2019 (−0,8%, Figure 1A).

The number of drug prescriptions during the pandemic was less variable than the frequency of consultations. Prescriptions decreased massively during the 1st wave in May 2020 (−5,4%). They increased slowly to an even higher number of prescriptions compared to the pre-pandemic times (+1,1%), with only slight decreases in prescriptions during each wave (Figure 1B).

The number of hospital admissions was below pre-pandemic levels throughout the observer period with the largest decrease during the 1st wave (−30,3%) and the summer plateau of 2021 (−3,5%; Figure 1C).

The cancer diagnoses (ICD C00-C99) decreased rapidly during the pandemic and did not reach pre-pandemic levels throughout the study period (Figure 1D).

### 3.2 Changes in practice management and reasons for the decline in incidence

Approximately one-third of all urologists reduced domiciliary visits (32%), visits to nursing homes (25%), number of consultations (35%) as well as consultations hours (20%) during the pandemic. Urologists reported less reduced domiciliary visits than general physicians and other specialists (25% vs. 39%, *p* = 0.025; Table 1). Consultations of high-risk patients were also reduced by 71%. 61 and 90% of all urologists reported that urgent and elective surgeries were difficult to organize, even more often than non-urologists (61% vs. 42%, *p* = 0.003; 90% vs. 66%, *p* < 0.001). Compared to pre-pandemic levels in 2019, preventive medical examinations and elective consultations were canceled in 40 and 36%, respectively. Telemedical approaches (telephone consultation, videotelephony) were used in 58%. Almost 60% of all urologists reported COVID-19-related absences of employees, and one in four practices had to close temporarily during the pandemic.

**Table 1:** Changes in practice management, displayed as a comparison of urologists and the whole study population (general physicians and other specialists except urologists).

**Parameter**	**Study population** (*n* = max 463)	**Urologists** (*n* = max 87)	*p* value
Question: this statement applies to my practice …			
Reduced domiciliary visits	39%	25%	0.025
Reduced visits of nursing homes	43%	32%	0.058
Reduced number of consultations	41%	35%	0.324
Reduced consultation hours	17%	20%	0.520
Reduced consultation of high-risk patients	71%	71%	1.000
Reduced use of external diagnostics due to risk of infection	10%	9%	0.840
Complicated organization of urgent surgeries	42%	61%	0.003
Complicated organization of elective surgeries	66%	90%	<0.001
Question: Changes during the COVID-19 pandemic compared to 2019…			
Reduced domiciliary visits	42%	30%	0.075
Reduced visits of nursing homes	44%	33%	0.140
Cancelation of preventive medical examinations	38%	40%	0.803
Cancelation of elective consultations	44%	36%	0.218
Use of telemedical approaches (telephone consultation, videotelephony)	70%	58%	0.098
Closure of practice	23%	25%	0.749
Absence of employees	58%	59%	0.900
Shortage of consumables	78%	71%	0.188
Separate waiting area inside the practice	42%	31%	0.073
Separate waiting area outside the practice	61%	66%	0.472
COVID-19 hygiene concept	88%	85%	0.477
Separation of patient flows (triage)	44%	25%	0.001
Use of ventilation system	43%	41%	0.906
COVID-19 training of stuff	75%	77%	0.787

### 3.3 Urologists perception of the reasons for the reduced consultations and recognition of incident diagnoses

87% of all urologists noticed a decreased consultation rate during the first wave of the pandemic. 95% of all urologists stated that patients’ behavior is the most important reason for reduced consultations (Table 2). Most urologists (88%) fear that the reduced detection rate of incident disease will harm patients’ outcomes. 53% of all urologists noticed the missing rise of consultations during the subsequent waves of the pandemic. The main reasons were still seen in patients’ behavior (85%) and persistent stress of the pandemic (59%).

**Table 2:** Perception of the reasons for the reduced consultations and incidences of diagnosis, as described by German urologists.

**Parameter**	**Urologists** (*n* = max 73)
Reduced consultations during the first wave of the COVID-19 pandemic Noticed by urologists Reasons patients’ behavior management of practice	87% 95% 39%
Reduced number of new diagnoses during the first wave of the COVID-19 pandemic Noticed by urologists Reasons patients’ behavior postponements of appointments by patients management of practice COVID-19 measurements Use of telemedical approaches Will have negative effect on patients’ outcome	53% 92% 81% 26% 31% 48% 88%
Missing rise of consultations during the subsequent waves of the COVID-19 pandemic Noticed by urologists Reasons patients’ behavior management of practice persistent stress additional services	53% 85% 22% 59% 38%

### 3.4 Level of anxiety, stress, and the need for support during the pandemic

Urologists reported the highest level of anxiety during the first lockdown (Mean 6.1 (SD 2.5), Table 3). The level of anxiety slowly decreased until it increased moderately again in the fourth wave (Mean 4.4 (3.1)). Except for the fourth wave, urologists reported a significantly higher anxiety level than general physicians and other specialists (1^st^ wave: *p* = 0.010; 2^nd^ wave: *p* = 0.036; 3^rd^ wave: *p* = 0.009; 4^th^ wave: *p* = 0.121) and a higher stress level and need for support during the pandemic than in the pre-pandemic period.

**Table 3:** Anxiety, stress and need for support during the pandemic, displayed as a comparison of urologists and the whole study population (general physicians and other specialists except urologists).

**Level of anxiety**			
**Time point**	**Study population** (*n* = max 483)	**Urologists** (*n* = max. 90)	*p* value
Pre-pandemic period (2019-March 2020)	2.3 (2.5)	3.1 (2.8)	0.009
1^st^ wave (March 2020–June 2020)	5.3 (2.8)	6.1 (2.5)	0.010
Summer plateau +2^nd^ wave (July 2020-Febuary 2021)	4.5 (2.8)	5.2 (2.8)	0.036
3^rd^ wave (March 2021–June 2021)	3.5 (2.6)	4.3 (2.9)	0.009
4^th^ wave (August 2021–December 2021)	3.9 (2.9)	4.4 (3.1)	0.121
**Level of stress**			
**Time point**	**Study population** (n = max 481)	**Urologists** (n = max 89)	**value of p**
Pre-pandemic period (2019-March 2020)	4.8 (2.4)	5.4 (2.3)	0.022
1^st^ wave (March 2020–June 2020)	6.5 (2.5)	6.9 (2.2)	0.189
Summer plateau +2^nd^ wave (July 2020-Febuary 2021)	6.3 (2.4)	6.6 (2.3)	0.298
3^rd^ wave (March 2021–June 2021)	6.7 (2.6)	6.7 (2.6)	0.888
4^th^ wave (August 2021–December 2021)	7.1 (2.6)	7.1 (2.5)	0.809
**Level of need for support**			
**Time point**	**Study population** (n = max 461)	**Urologists** (n = max 86)	*p* value
Pre-pandemic period (2019-March 2020)	2.9 (2.7)	3.4 (2.7)	0.103
1^st^ wave (March 2020–June 2020)	5.1 (3.0)	5.6 (3.0)	0.187
Summer plateau +2^nd^ wave (July 2020-Febuary 2021)	4.7 (2.9)	5.0 (2.9)	0.333
3^rd^ wave (March 2021–June 2021)	4.9 (3.1)	4.9 (3.0)	0.898
4^th^ wave (August 2021–December 2021)	5.0 (3.2)	5.1 (3.0)	0.884

Values are displayed as mean (SD) and range 0–10.

Multivariate analysis revealed a significantly higher level of anxiety in female urologists during the fourth wave (*p* = 0.016; Supplementary Table S2). Regarding the level of stress, urologists treating a higher number of patients (>1,500 patients/quarter) reported a higher level of stress in the third wave (*p* = 0.038) and in the fourth wave (*p* = 0.043). In addition, urologists in individual practices described a higher stress level in the fourth wave than urologists in community health centers (*p* = 0.018).

Reasons for anxiety and stress were mostly patient behavior (48.4%) and organization of practice (18.7%) in the first wave (Supplementary Table S3). In the second wave, private burdens had growing importance (20.0%). In the further course, increasing bureaucracy (third wave: 21.4%; fourth wave: 26.4%) as well as additional services (third wave: 29.2%, fourth wave: 17.1%), such as vaccination or COVID-19 testing, were the most important reasons. However, patient behavior still represented an important factor.

## 4 Discussion

Our study was the first to evaluate the long-term effect of the COVID-19 pandemic on work-related and personal aspects among urologists in the German ambulatory sector. The decline in consultations, drug prescriptions, hospital admissions, and cancer diagnosis detection during the pandemic was in line with previous studies, showing a significantly decreased number of cancer screenings and diagnoses during the early phase of the COVID-19 pandemic (15).

However, data evaluating its longitudinal change was lacking. A German survey assessed the provision of medical care services during the first and second waves of the COVID-19 pandemic (16). Congruent to our data, the urologic outpatient sector described a 50% reduction in consultation during the first wave. This is also in line with studies reporting a strong impact of the pandemic on German urologists’ daily work (8). An international cohort study with more than 20,000 cancer patients confirmed that cancer surgery systems were worldwide fragile to lockdowns, as 14% of all patients awaiting surgery did not undergo planned surgery and experienced longer preoperative delays during lockdowns (17). This is in line with the high number of cancelations of preventive medical examinations (40%) in our study. Our study revealed that consultations, hospital admissions, and cancer diagnoses decreased massively, not reaching pre-pandemic levels throughout the study period. A previously published analysis supports our findings regarding prostate cancer, as fewer prostate cancer patients were surgically treated during the first two waves of the pandemic, which did not reach pre-pandemic levels (18).

Nine out of ten urologists in our study fear that the reduced detection rate of incident disease will harm patients’ outcomes. French studies aroused suspicion that a reduced consultation and detection rate of cancer diagnoses during the pandemic might lead to a higher tumor burden, number of advanced tumors (pT3b: 11.2 vs. 25.6%; nodal positive: 14.8 vs. 46.1%) and metastatic disease (5.9 vs. 9.3%) of prostate cancer patients (19, 20). However, long-term data is missing so far but is urgently needed. Thus, the fine line between a shutdown and a potential negative impact on the healthcare system should be intensively evaluated in future pandemic waves.

During the pandemic’s beginning, German urologists awarded telehealth for having a high relevance. Still, only 25,5% of all urologists from the ambulatory sector already included telemedical approaches in their daily routine (8). In the survey two years later, telemedical approaches were used by 58% of all urologists from the outpatient sector, showing that telemedicine has steply risen during the pandemic (5). Although telemedicine incorporates advantages in a pandemic, our study participants stated that it might have complicated the identification of incident diagnoses, indicating a loss of vital clinical information and that physical examination and face-to-face consultations might not be replaceable. Consequently, healthcare professionals and patients must be trained in telemedical approaches before widespread uptake, and accurate electronic patient notes must be available. Especially follow-up consultations seem to be ideal for telemedical procedures (21).

Almost 60% of all urologists reported COVID-19-related absences from employees. One in four practices had to close temporarily during the pandemic, underlining the COVID-19 pandemic impact on health systems and social and economic structures (22). The observations of the pandemic’s influence can be used as a call for dynamic systemic transformation and improved resilience of healthcare workers. The World Health Organization also expounds on the potential negative impact of the COVID-19 pandemic on mental health of healthcare professionals, strongly impacting the work-life balance (12). In our study, urologists reported higher anxiety, stress, and need for support during the pandemic than in the pre-pandemic period. Anxiety and the need for support were highest during the first wave of the pandemic, which is in line with prior studies from China and Europe performed at the beginning of the pandemic revealed a high psychological burden among healthcare workers, especially in the outpatient sector (8, 23–26).

Our analysis revealed that urologists in individual practices and those treating more patients experienced the highest stress level in the third and/or fourth waves. The higher anxiety level in female urologists during the fourth wave is in line with the results of a German and French survey that evaluated mental health issues in healthcare workers during the beginning of the pandemic (8, 27). However, a previous analysis examining the pandemic-related stress experience of psychiatrists revealed that anxiety was dependent on feeling restricted (OR = 5.52) and risk of infection (OR = 5.74) but not on gender (28). The physical and psychological burden healthcare workers experienced during the COVID-19 pandemic is high, as the prevalence of burnout among physicians was nearly 60% (13). Further analysis of potential risk factors for developing mental health problems is paramount. Mental health and resilience could be supported by specific interventions and psychological support (29), which can be classified into four main categories: social/structural support, work environment, communication/information, and mental health support. However, systematic reviews revealed lacking evidence regarding selecting interventions beneficial to frontline workers’ resilience and mental health (29, 30). Future research is needed to promote mental well-being and resilience strategies in healthcare professionals during and after pandemics. Limitations of our study include the descriptive design of the analyses of consultations, drug prescriptions, hospital admission or incident cancer diagnoses over the pandemic periods, which did not account for the analyses of statistical significances. Furthermore, we used a non-validated survey, potentially leading to non-sampling errors which might negatively impact the accuracy and reliability of the results. Additionally, the low response rate of 5% of our survey could have negatively impacted the reliability and validity of the results.

## Data availability statement

The original contributions presented in the study are included in the article/Supplementary material, further inquiries can be directed to the corresponding author.

## Ethics statement

The survey was approved by the Ethical Committee of the Chamber of Physicians of Mecklenburg-Western Pomerania (registry number (BB 127/21) and was conducted in accordance with Good Clinical Practice Guidelines: all participants agreed on the conditions of the survey before taking part.

## Author contributions

PP: Data curation, Formal analysis, Writing – original draft, Writing – review & editing. MP: Formal analysis, Methodology, Writing – review & editing. KK: Formal analysis, Writing – review & editing. SL: Writing – review & editing. JB: Methodology, Writing – review & editing. BM: Formal analysis, Methodology, Writing – review & editing.
