# Supplementary material for: Medical care services provision and stress experience in urologists during all waves of the COVID-19 pandemic in Germany
Source: Front Med (Lausanne). 2024 Feb 9;11:1320489. doi: 10.3389/fmed.2024.1320489 (PMC10893761; doi:10.3389/fmed.2024.1320489)
Supplement: Supplementary file 1 [file Data_Sheet_1.docx]

# Supplementary material

## Study design

This study comprised a i) secondary data analysis based on medical record data from the Disease Analyzer database (IQVIA) to examine changes in healthcare service provision and ii) a survey to assess perceived reasons for these changes.

i) The secondary data captured consultations, drug prescriptions, specialist referrals, diagnoses made, and basic medical and demographic data directly and anonymously from the practices via an interface to their respective practice management software. The analysis was based on data from a total of 6.1 million treatment cases per year (patients aged 18 years and older) documented by 996 GPs and internists or 798 specialist practices (n = 224 gynecologists, n = 147 orthopedists, n = 127 neurologists and psychiatrists, n = 133 ear, nose and throat (ENT) physicians, n = 83 dermatologists, and n = 84 urologists) in Germany between September 2019 and February 2020 (pre-pandemic period) as well as March 2020 and September 2021 (pandemic period). The Disease Analyzer database (IQVIA) was used for secondary data as published elsewhere [1]. The main outcomes were the following: the number of (1) GPs and specialist consultations (telephone contacts and visits), (2) hospital admissions, (3) specialist referrals, and (4) recognized diseases. The ICD-10 diagnoses C00–C99 were used to represent the recognition of cancer. Healthcare utilization and disease recognition were presented as frequencies whereby the pandemic periods (2020 and 2021) were compared with the corresponding prepandemic periods in 2019. For this purpose, percentage differences were calculated for both the individual waves and overall pandemic course compared to the respective prepandemic period to assess the change during the COVID-19 pandemic in Germany.

ii) The anonymous online survey was generated based on the following steps: (i) review of the relevant literature, (ii) development of the first draft of the questionnaire by a previously formed core team of three researchers, (iii) consultation of the questionnaire with representatives (i.e. experts) of all professional associations as well as (iv) finalization and preparation for distribution. The questionnaire was distributed using the cloud-based open-source tool LimeSurvey. The Professional Association of German Urologists e.V shared the survey link between 04 December 2021 and 28 February 2022 with their members via different communication channels. In total, n = 101 urologists responded to the online survey questionnaire. The survey as demonstrated in the supplementary materials covered the following outcomes next to physicians demographic and practices characteristics: (1) perceived causes for the decreased and non-compensated number of consultations and recognized diseases during the 1st-COVID-19-wave and the following waves, and (2) the changes made in practice management to reduce infection risk. To assess physicians' perceptions in more detail, two to five additional items assess on a 5-Point-Likert-type response scale (1= "does not apply" to 5= "applies") whether the physicians thought that themselves or the patient caused the decrease or lack of compensation. Changes in practice management comprise eight items assessing whether the practices changed their management (1= "does not apply" to 5= "applies") and 11 items assessing how often items were performed compared to pre-pandemic (1= "much rarer" to 5= "much more frequently"). Each of the six items represented measures for infection risk reduction and was assessed dichotomously (yes vs. no). The survey was approved by the Ethical Committee of the Chamber of Physicians of Mecklenburg-Western Pomerania [registry number (BB 127/21)].

## Statistical analyses

Descriptive statistics were used to demonstrate changes in healthcare provision, recognition of cancer cases and perception and views on causes of these changes. Fisher's exact Tests were used to check significance of these differences. Multivariate regression models were used to assess associated factors of stress, anxiety and support needed of urologists. Analyses were performed using SAS version 9.4 (Cary, NC: SAS Institute Inc) and STATA/IC 16.

## Observation period

Within our study, we use the classification system published by the Robert Koch Institute (RKI), Germany's national public health institute, which retrospectively divides the COVID-19 pandemic into several waves [2]: 1st-COVID-19-Wave (March–May 2020), summer plateau 2020 (June–September 2020), 2nd-COVID-19-Wave (October 2020–February 2021) 3rd-COVID-19-Wave (March–June 2021), summer plateau 2021 (June–July 2021) and 4th-COVID-19-Wave (August–September 2021).

## References

[1] Rathmann W, Bongaerts B, Carius HJ, Kruppert S, Kostev K. Basic characteristics and representativeness of the German Disease Analyzer database. Int J Clin Pharmacol Ther 2018;56:459–66. https://doi.org/10.5414/CP203320.

[2] Schilling J, Buda S TK. Zweite Aktualisierung der „Retrospektiven Phaseneinteilung der COVID-19- Pandemie in Deutschland“. Epid Bull 2022;103-5 n.d. https://doi.org/10.25646/9787.

**Supplementary table 1**: Characteristics of study population

| **Parameter** | **Total (n, %)** |
| --- | --- |
| **Number of participants** | n = 101 |
| **Age**  <35 years  35-44 years  45-66 years  >65 years | -  8 (8%)  84 (83%)  9 (9%) |
| **Gender**  Female  Male | 12 (12%)  89 (88%) |
| **Employment**  Self-employed  Employed | 99 (98%)  2 (2%) |
| **Number of patients per quarter**  <1000  1001-1500  >1500 | 10 (10%)  30 (30%)  61 (60%) |
| **Contact with designated COVID-19 patients**  Never  Rarely  Often | 9 (9%)  81 (80%)  11 (11%) |

**Supplementary table 2**: Multivariate logistic regression analysis for the change of perceived burden (change in anxiety, distress, and support needs) during the pandemic in urologists (max. n=90). The model was adjusted for the following covariables: sociodemographic characteristics of the physicians (age, sex) and characteristics of their practices (employment (self-employed vs. employed), outpatient facility (individual practice vs. community health center), diagnostic alignment (yes vs. no), number of patients per quarter (0 – 1.000 vs. 1.001 – 1.500 vs. > 1.500) and contact with designated COVID-19 patients (never vs. rarely vs. often vs. very often)).

| **Parameter** | ***b*** | **95% Confidence Interval** | **p-value** |
| --- | --- | --- | --- |
| Level of anxiety in female urologists | | | |
| 1^st^ wave | 1.455 | -0.070-2.980 | 0.061 |
| 2^nd^ wave | 0.528 | -1.121-2.177 | 0.526 |
| 3^rd^ wave | 1.058 | -0.531-2.646 | 0.188 |
| 4^th^ wave | 2.211 | 0.420-4.002 | 0.016 |
| Level of stress depending on the number of patients | | | |
| 1^st^ wave  1001-1500 patients/quarter  >1500 patients/quarter | -1.391  -1.764 | -3.245-0.464  -3.701-0.178 | 0.139  0.074 |
| 2^nd^ wave  1001-1500 patients/quarter  >1500 patients/quarter | 0.981  0.893 | -0.853-2.816  -1.014-2.800 | 0.290  0.354 |
| 3^rd^ wave  1001-1500 patients/quarter  >1500 patients/quarter | 1.454  2.185 | -0.552-3.461  0.123-4.249 | 0.153  0.038 |
| 4^th^ wave  1001-1500 patients/quarter  >1500 patients/quarter | 0.869  2.071 | -1.057-2.797  0.067-4.074 | 0.371  0.043 |
| Level of stress in urologists in community health centers (vs. individual practices) | | | |
| 1^st^ wave | 0.010 | -1.127-1.147 | 0.986 |
| 2^nd^ wave | -0.334 | -1.431-0.763 | 0.547 |
| 3^rd^ wave | -1.016 | -2.204-1.723 | 0.093 |
| 4^th^ wave | -1.396 | -2.550-(-)0.242 | 0.018 |

**Supplementary table 3**: Reasons for anxiety and stress during the pandemic.

| **Parameter** | **1^st^ wave** (n, %)  (Participants: n=91) | **2^nd^ wave** (n, %) (Participants: n=90) | **3^rd^ wave** (n, %) (Participants: n=89) | **4^th^ wave** (n, %) (Participants: n=88) |
| --- | --- | --- | --- | --- |
| Additional services | - | 4 (4.4%) | 26 (29.2%) | 15 (17.1%) |
| Patient behavior | 44 (48.4%) | 15 (16.7%) | 11 (12.4%) | 14 (15.9%) |
| Organization of practice | 17 (18.7%) | 24 (26.7%) | 11 (12.4%) | 6 (6.8%) |
| rivate burdens | 7 (7.7%) | 18 (20.0%) | 4 (4.5%) | 9 (10.2%) |
| Maintenance of care | 5 (5.5%) | 6 (6.7%) | 9 (10.1%) | 11 (12.5%) |
| Financial burdens | 8 (8.8%) | 7 (7.8%) | 7 (7.9%) | 7 (8.0%) |
| Bureaucracy | 9 (9.9%) | 11 (12.2%) | 19 (21.4%) | 23 (26.4%) |
| Others | 1 (1.1%) | 5 (5.6%) | 2 (2.2%) | 3 (3.4%) |
